# Excision of the Elbow Joint

**Published:** 1830

**Authors:** 


### II. Analytical.

1. *Excision of the Elbow Joint.*—This operation has lately been twice performed by Mr. Jas. Syme, of the Edinburgh Surgical Hospital. Both patients laboured under well marked caries of the joint. In the first patient, a young woman aged 25, the cure was delayed by a chronic bronchitis. She left the Hospital considerably better in this respect than when she entered, and with a prospect of retaining a useful arm.

The boy, aged 9, was a very favorable subject for the operation. After sawing off the humerus, and cutting away with the pliers, the olecranon and head of the radius, the cut extremity of the olecranon was found to be carious. Having ascertained the depth of the caries by means of a probe, the ulna was separated from its connexions as far as was necessary, and cut across. The whole spongy portion of the bone was then removed, although with some difficulty, owing to the connexion of the brachiæ us internus. Although the biceps was the only muscle whose connections were undisturbed, yet in five weeks he began to recover the motion of the arm which was nearly as moveable as ever. As the students found it difficult to conceive how the use of the muscles could be recovered, Mr. S. shewed them a young clergyman, upon whom he had operated some years before, who could not only write sermons, but execute all the ordinary motions of the arm.—*Medico Chirurgical Rev.*

